# Preparation of porous bio-char and activated carbon from rice husk by leaching ash and chemical activation

**DOI:** 10.1186/s40064-016-2932-8

**Published:** 2016-08-03

**Authors:** Md. Ahiduzzaman, A. K. M. Sadrul Islam

**Affiliations:** 1 Department of Agro-processing, Bangabandhu Sheikh Mujibur Rahman Agricultural University, Salna, Gazipur, 1706 Bangladesh; 2 Department of Mechanical and Chemical Engineering, Islamic University of Technology, Gazipur, 1704 Bangladesh

**Keywords:** Rice husk char, Porous bio-char, Activated carbon, Surface area, Adsorption kinetics, Adsorption isotherm

## Abstract

Preparation porous bio-char and activated carbon from rice husk char study has been conducted in this study. Rice husk char contains high amount silica that retards the porousness of bio-char. Porousness of rice husk char could be enhanced by removing the silica from char and applying heat at high temperature. Furthermore, the char is activated by using chemical activation under high temperature. In this study no inert media is used. The study is conducted at low oxygen environment by applying biomass for consuming oxygen inside reactor and double crucible method (one crucible inside another) is applied to prevent intrusion of oxygen into the char. The study results shows that porous carbon is prepared successfully without using any inert media. The adsorption capacity of material increased due to removal of silica and due to the activation with zinc chloride compared to using raw rice husk char. The surface area of porous carbon and activated carbon are found to be 28, 331 and 645 m^2^ g^−1^ for raw rice husk char, silica removed rice husk char and zinc chloride activated rice husk char, respectively. It is concluded from this study that porous bio-char and activated carbon could be prepared in normal environmental conditions instead of inert media. This study shows a method and possibility of activated carbon from agro-waste, and it could be scaled up for commercial production.

## Background

Activated carbon is highly porous in its structure, non-hazardous, carbonaceous material having a large internal surface area. The activated carbon can adsorb a wide variety of substances including different types of dyes and heavy metals. It is capable to attract molecules to internal surface and is therefore, called adsorbent. It, also called activated charcoal or activated coal, is a form of carbon that has been processed to make it extremely porous and thus to have a very large surface area available for adsorption or chemical reactions. One of the main characteristics of activated carbon is their adsorption capacity. It is the porous structure and chemical nature of the surface of active carbons that govern this property, both of which are related to the crystalline composition.

Textile and dyeing industries produces huge polluted waste water and drains into natural water shade, canals, rivers and lagoons etc. The dyes are pollutants of high environmental impact, because of their widespread use and their potential to toxic aromatic amines accounting approximately 50 % of worldwide production (Guettai and Amar 2005; Rys and Zollinger 1992). About 20 % of synthetic dyes are lost in the waste water stream that is also important source of pollution (Zollinger 1991). Due to the release of dyes in aquatic ecosystem a dramatic consequences caused through the toxicity, aesthetic pollution and perturbations in aquatic life. Since the modern dyes are stable in aquatic system, biological treatments for eradication of textile and dyeing effluents are ineffective (Dai et al. 1995, 1996; Chung et al. 1981). Activated carbon derived from alkali treated rice husk indicated good adsorption capacity for methylene blue in aqueous solutions (Lin et al. 2013), and for nitrogen adsorption/desorption (Liu et al. 2016). Activated carbon has several important usages including solution purification, removal of tastes and odors from domestic and industrial water supplies, vegetable and animal fats and oils, alcoholic beverages, chemicals and pharmaceuticals and in the waste water treatment. It is a versatile product with good market demand. The non-availability of high quality activated carbon in the Bangladesh market, to cater to the needs of the pharmaceutical and fine chemical sector has necessitated imports into Bangladesh. Despite its prolific use of activated carbons in water and wastewater industries, commercial activated carbons remain an expensive material. This has led to a search for low-cost, easily available materials as alternative adsorbent materials. Proper utilization of agro industrial by-product is very much important for economy of a nation. A wide variety of carbons have been prepared from agricultural waste such as coconut shells (Laine et al. 1989), cotton stalk (Grigis and Ishak 1999), sugarcane bagasse (Ahmedna et al. 2000), coir pith (Kadirvelu et al. 2003), straw (Namila and Mungoor 1993), rice husk (Lin et al. 2013; Liu et al. 2016). Each adsorbent has its drawbacks and advantages. Previous study reported that significant changes observed in activated carbon with gradually increased in activation temperature ranging from 600 to 800 °C (Lin et al. 2013).

Inert media (nitrogen) is commonly used for activation of carbon at high temperature in the range of 600 to 900 °C (Ahmedna et al. 2000; Shimda et al. 1999; Guo et al. 2008; Lin et al. 2013; Liu et al. 2016). Activated carbon preparation under a nitrogen medial adds cost of production of activated carbon and also complex experimental setup. The present work demonstrates the feasibility of activated carbon preparation form of rice husk locally available as low-cost adsorbent materials at normal environmental condition.

## Experimental procedures

### Char production

Rice husk is washed thoroughly with tap water initially to remove mud and other water soluble impurities and then the materials are washed with distilled water to remove other impurities. After washing the materials are dried in an oven at 105 °C for 24 h. The samples are preserved in desiccators to avoid further absorption of moisture. Dried rice husk sample is taken in a porcelain crucible and covered with lid and placed in a proportional–integral–derivative (PID) control muffle furnace at 650 °C for an hour. The carbonized husk samples are cooled and preserved for using for the next step of the procedure.

### Experimental treatments for preparation of activated carbon

The aim of the different treatments for preparation of activated carbon is to identify the best way of producing quality activated carbon. In this context, a single variety of rice husk is taken to investigate the activated carbon preparation with a temperature level 600, 700, 800 and 900 °C. Heating duration ranges from 30 to 120 min with a 30 min interval. As ash in rice husk char retards its’ porousness, it is treated with alkali solution (sodium hydroxide) for leaching ash from it. After removal of ash from rice husk char, the char is washed and dried in oven and kept in desiccators for next use. The treated rice husk char then activated with zinc chloride. The chemicals are used at different ratio level of char and chemical at different temperature level.

### Activated carbon preparation

#### Oxygen-cut down environment by double crucible method

In general activated carbon is prepared in an inert media (nitrogen or argon). However the preparation of inner environment for activated carbon is little bit inaccessible in general at local condition. Then an alternative method was applied in this study called double crucible method. In this method the char was placed in a smaller silicon crucible covered with ground silicon lid (Tatlok brand). The small crucible containing rice husk char is put in a bigger porcelain crucible. The gap inside the bigger crucible is filled up by raw husk to make reduce oxygen environment inside the crucible and finally the bigger crucible was covered with lid. In this method, the bulk volume of air inside the bigger crucible is replaced by raw char, again when the crucible is heated up; firstly the volume of air inside the crucible is expanded and a portion of the air comes out from both the small and bigger crucible; secondly the volatile part of rice husk is reacted with oxygen and it is assumed that the entire amount of oxygen is exhausted from the crucible during the heating process. Since there is no inert media is used in this study it will be cost effective process.

#### Chemical activation of rice husk char

Two grams (2 g) of rice husk char was soaked in zinc chloride solution for 24 h. Then the crucible was placed inside a muffle furnace to give a required heat treatment. After activation with zinc chloride, the samples are washed with 0.3 (N) hydrochloric acid solutions firstly and then washed with distilled water until the pH value reached 7.0. After washing the samples are dried in an oven at 105 °C for 24 h. Then the dried samples are preserved in desiccators to avoid further absorption of moisture.

### Characterization of the activated carbon

The activated carbon is firstly evaluated with methylene blue (MB) dye adsorption test. One of the main purposes of this study is to find out the best combination of activation factors (precursor, temperature, activation agent, degree of heat treatment) to obtain best product from rice husk. Therefore, several steps and procedures are followed successively to reach the ultimate goal.

The effect of temperature on activation of rice husk char was studied. Three levels of temperature viz. 600, 700 and 800 °C are employed for 2 h of heating for each level. The heat treated rice husk chars are then evaluated by adsorbing the methylene blue. Rice husk char (0.05 g of each sample) is weighed by a 4-decimal digital balance (Model: OHAUS) and mixed in 100 mL of 10^−4^ M MB solution. The mixture of dye and char is then stirred and kept for about 7 h. After adsorption the dye solution is taken into a centrifuge tube. The dye solution is then centrifuged for 20 min at 1500 rpm to settle down the char particle at the bottom of the tube. Clean solution is taken from upper part of the tube. Then the solution is put in Vis UV equipment to measure the maximum absorbance at 664 nm of wave length.

MB adsorbed is measured by using visible ultra violet (Vis UV) equipment (model 6715 UV/Vis spectrophotometer, JENWAY) in the Environmental Engineering Lab of Civil and Environmental Engineering, Dept of Islamic University of Technology. A calibration chart of MB with respect to absorbance of ultra-violet (UV) is determined. Coefficient of extinction is found to be 65,280 L mole^−1^ cm^−1^.

The concentration of MB after adsorption is determined using the following equation:

1 $$C_{e} \, = \,\frac{A}{E}$$

where C_e_ = concentration of MB solution after adsorption; A = absorbance of Vis UV, cm^−1^; E = coefficient of extinction, 65,280 L M^−1^ cm^−1^.Then the amount of MB adsorbed is calculated by using the following equation:

2 $$q_{e} \, = \,\frac{{(C_{0} \, - \,C_{e} )V}}{m}\, \times \,W$$

where q_e_ = uptake of dye by adsorbent, mg g^−1^; C_0_ = initial concentration of dye, M L^−1^; C_e_ = final concentration of dye, M; V = volume of dye solution, L; m = weight of activated carbon, g; W = mole weight of MB (319.86 × 1000), mg/mole.

### Specific surface area determination by MB adsorption

Specific surface area is calculated from the amount of MB adsorbed. The occupied surface area by one molecule of MB is considered to be 130 **Ǻ**^2^. Then the specific surface area is calculated by using the following equation:

3 $$S_{s} \, = \,\frac{{q_{e} \, \times A_{V} \, \times \,A_{MB} }}{{W\, \times \,10^{ 20} }}$$

where S_s_ = specific surface area, m^2^g^−1^; q_e_ = amount of MB adsorbed, mg g^−1^; W = molecular weight MB, mg/mole; A_V_ = Avogadro’s number (6.02 × 10^23^ per mole); A_MB_ = area covered by one molecule of MB (130 Ǻ^2^).

### Contact time study

To determine the equilibrium time a kinetic study is carried out. The time to reach equilibrium is determined by a series of measurements over the range of 30–480 min at room temperature.

### Adsorption kinetics study

To study the adsorption kinetics of activated carbon three different models are studied. The models are (1) pseudo-first order kinetic model, (2) pseudo-second order kinetic model and (3) intra particular diffusion model.

### Adsorbent dosage study

The effect of adsorbent dosages on the equilibrium adsorption of MB in solution is studied. In this experiment the initial concentration of dye is used as 10^−4^ M (32 mg L^−1^). The dosages of activated carbon are used in the range of 0.01–0.035 g for each sample. The solution is stirred with a magnetic stirrer and kept the solution until the equilibrium of adsorption. The amount of dye adsorbed in the mg g^−1^ at equilibrium is calculated using Eq. 2.

### Adsorption isotherm model study

To study the adsorption isotherm models, three different models are studied. The models are (1) Freundlich isotherm model, (2) Langmuir isotherm model, and (3) Langmuir–Hinshelwood isotherm model.

## Results and discussion

### Effect of temperature on raw rice husk char

**Fig. 1:**
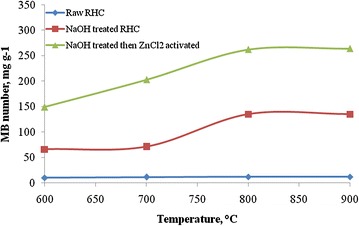
Effect of preparation temperature of activated carbon on MB adsorption

The MB number ranged from 10 to 12 mg g^−1^ (Fig. 1) and it decreases in trends with respect to increase in temperature. The decreasing trends might be due to the increase in ash content of rice husk char with increase in temperature. The specific surface area ranges from 25 to 28 m^2^ g^−1^.

### Effect of temperature on sodium hydroxide treated rice husk char

Ash in the rice husk char is removed by dissolving it in the sodium hydroxide solution. The ash content of rice husk char is lowered as low as 4 % from 54 % at initial condition. After removal of ash from rice husk char, 2.0 g of char is heated at 600, 700 and 800 °C of temperature and left for 2 h for each sample. After activation the samples are cooled and evaluation tests are done. The results showed that the adsorption of MB increased significantly compared to the previous results. The MB numbers are found to be 66, 71 and 135 mg g^−1^ at 600, 700 and 800 °C, respectively (Fig. 1). Corresponding values of specific surface area are 162, 174 and 331 m^2^ g^−1^.

### Effect of temperature on zinc chloride impregnated activated carbon

The ratio of zinc chloride and rice husk char is used 3:1 (Ahiduzzaman 2011). Four different temperature regimes are selected viz. 600, 700, 800 and 900 °C. The MB numbers are found to be 149, 203, 262 and 264 mg g^−1^ at the temperature of 600, 700, 800 and 900 °C, respectively (Fig. 1). The MB number increased with increase in temperature significantly in the range of 600–800 °C. The increase in MB number does not increase significantly between 800 and 900 °C temperature regimes. The specific surface area ranges from 365 to 645 m^2^ g^−1^ in the temperature regime of 600 to 900 °C. The specific surface area does not varied significantly beyond the temperature regime 800–900 °C. The duration of heat treatment in this study is 1 h. Further study might need to examine the effect of duration of heat treatment for preparation of activated carbon.

Rice husk char contained more than 50 % of ash which creates problem for pore development in the activated carbon. After removing the ash from the husk char the porousness of char is increased significantly. After activation with chemical like zinc chloride the char develops more pores.

### Scanning electron microscopy (SEM) analysis

**Fig. 2:**
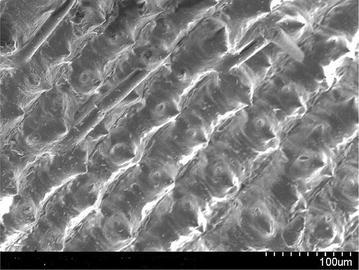
SEM image of raw rice husk

**Fig. 3:**
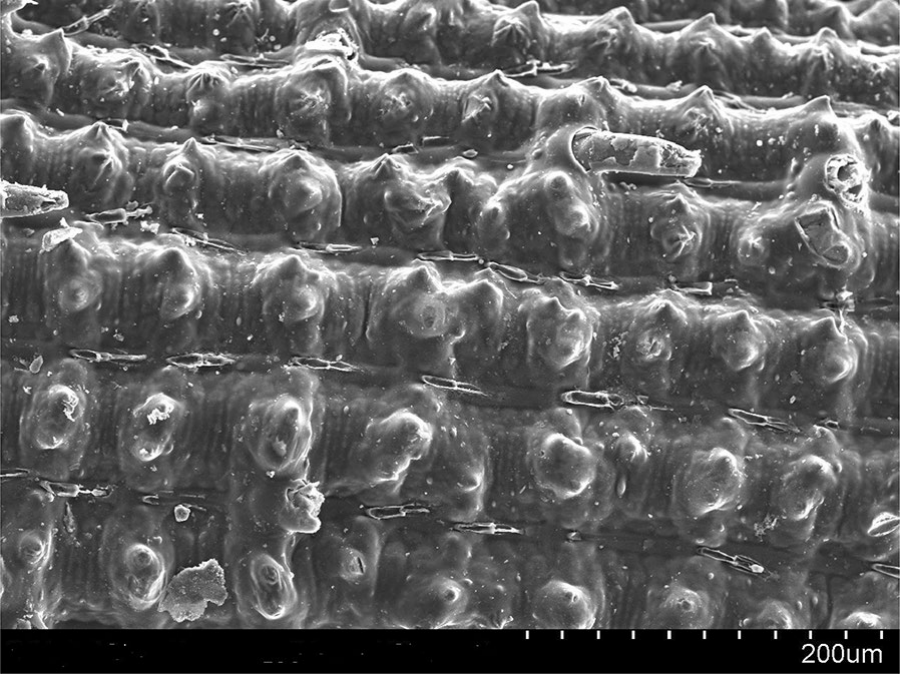
SEM image of rice husk char

**Fig. 4:**
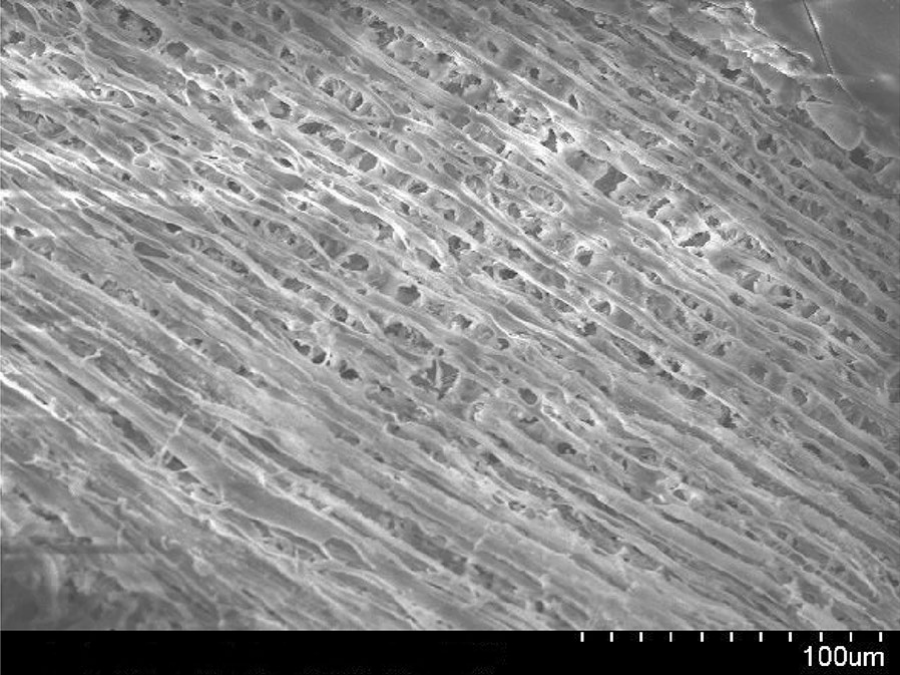
SEM image of rice husk ash

**Fig. 5:**
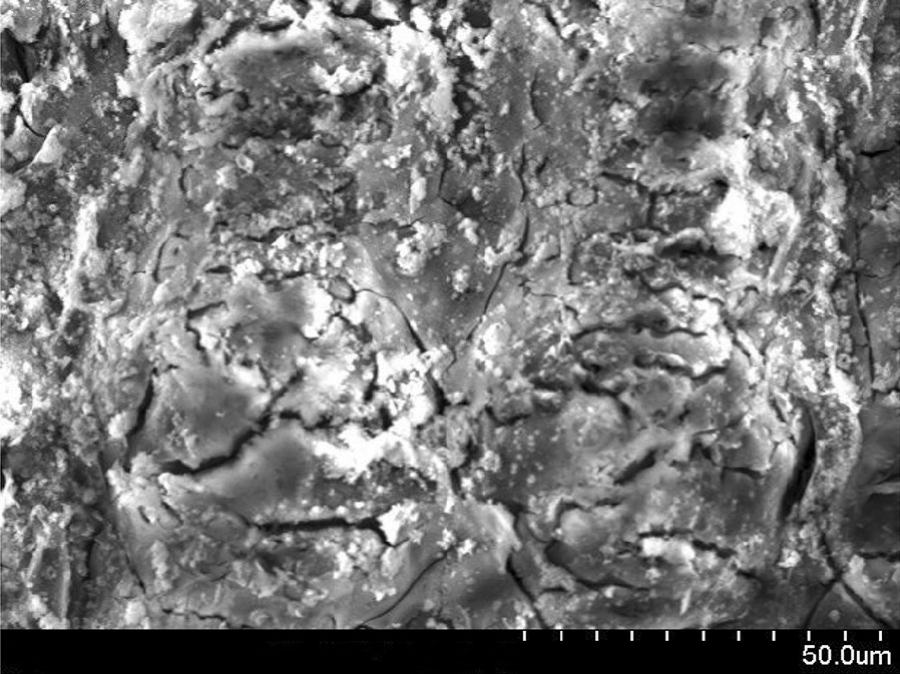
SEM image of NaOH treated rice husk char

**Fig. 6:**
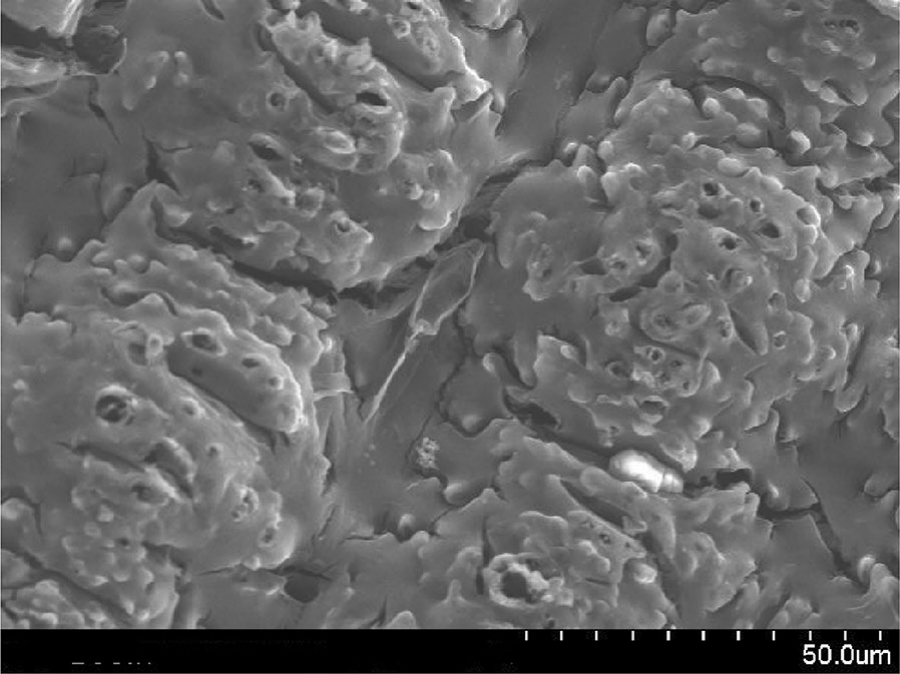
SEM image of activated rice husk char

Scanning electron microscope images of a sample is done by scanning it with a high-energy beam of electrons. The electrons interact with the atoms those make up the sample producing signals that contain information about the sample’s surface topography. In this study SEM images of raw rice husk, rice husk char, rice husk ash, rice husk char treated with sodium hydroxide and activated with zinc chloride. SEM image of activated carbon is taken by Hitachi N-3400 equipment at Bangladesh Council of Scientific and Industrial Research lab, Dhaka. SEM of raw rice husk and rice husk char shows surface topography without any pore (Figs. 2, 3). SEM image of rice husk ash indicates a porous structure in nature (Fig. 4) which means that after combustion organic compound including carbon is driven off and silica remains as its structure in the husk. In reverse, if the silica is driven off from the rice husk char by the treatment of sodium hydroxide then a porous carbon structure is obtained as shown in Fig. 5. For further development of porous structure, the sodium hydroxide treated rice husk is activated with zinc. The SEM image of zinc chloride treated activated carbon shows the well developed micro pore structure (Fig. 6).

### Effect of heating duration on zinc chloride impregnated activated carbon

**Fig. 7:**
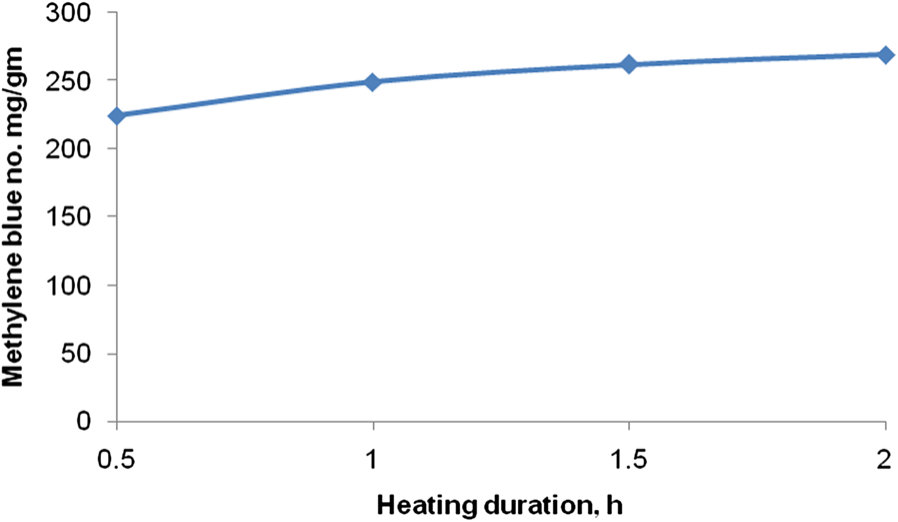
Effect of heating duration on the MB adsorption of activated carbon at 3.0 ratio of zinc chloride and rice husk char

From Fig. 1 it is clear that the highest MB number is found at the temperature of 800–900 °C regimes. Therefore, the temperature is selected 900 °C and the heating duration employed in the range of 30–120 min with an interval of 30 min. The MB number of the activated carbon increases with increase in heating duration. The MB numbers are found to be 224, 249, 262 and 269 mg g^−1^ for the heating duration of 0.5, 1.0, 1.5 and 2.0 h, respectively (Fig. 7). Corresponding specific surface area are found to be 548, 608, 649 and 659 m^2^ g^−1^ for the heating duration of 0.5, 1.0, 1.5 and 2.0 h, respectively.

### Contact time study

**Fig. 8:**
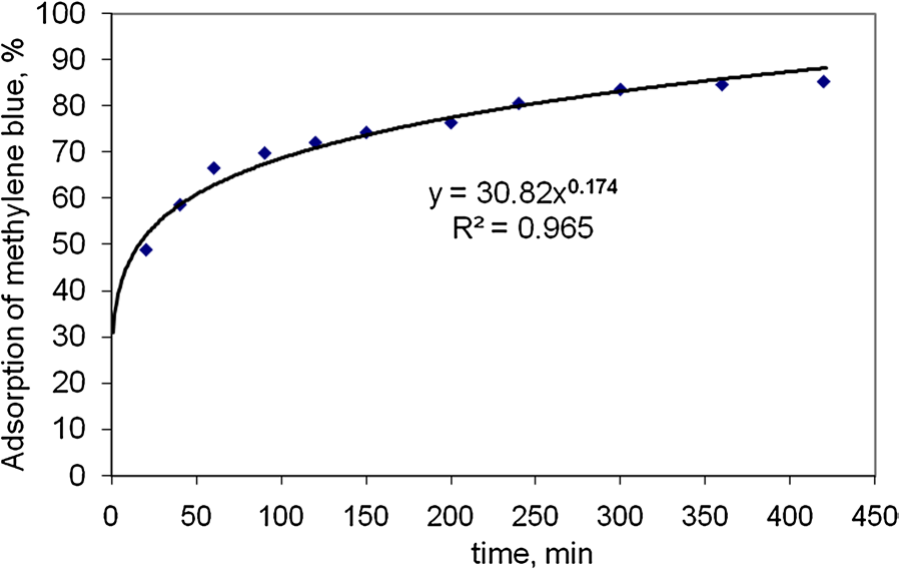
Kinetic curve adsorption of MB onto the activated carbon prepared from rice husk with zinc chloride activation

Contact time study examines the rate of adsorption by activated carbon prepared in this study. MB solution of 5 × 10^−5^ M (16 mg L^−1^) concentration is used in this investigation. 100 mL of MB solution is taken in reagent bottle and 0.01 g of activated carbon is mixed and stirred thoroughly. Sample solution is withdrawn at a predefined interval of time. The solution is centrifuged and carbon particle free solution is placed in a UV Vis spectrophotometer to determine the absorbance and finally to calculate the adsorbed amount of MB after a specific time. The kinetic curve of the activated carbon is shown in Fig. 8. The extent of MB dye removal by activated carbon increased with the increased of contact time. The removal percentage of dye is found to be rapid at the initial stage and becomes slower with the increase of contact time. This is due to the strong attractive forces between the dye molecules and the activated carbon.

### Adsorption kinetics

There are several kinetic models are proposed to illuminate the mechanism of solute adsorption by adsorbent. The analysis of adsorption dynamics illustrates the solute uptake rate and clearly controls the residence time of adsorbate uptake at the solid–liquid interface. In this investigation, the kinetics of MB adsorption on activated carbon is analyzed using three adsorption kinetic model. Those are pseudo first-order kinetic model of Lagergren (1898) (reported in Ho 2004), Pseudo second order kinetic model of Ho and McKay 2000 and intra-particle diffusion model of Weber and Morris (1963) (reported in Srivastava et al. 1989; Maiti 2007). The adsorption kinetic model analyzed using the adsorption data obtained from 0.01 g of different types of activated carbon dosage in 16 mg L^−1^ MB solution at predefined time interval up to 7 h of uptake time.

#### The pseudo first-order kinetic model

**Fig. 9:**
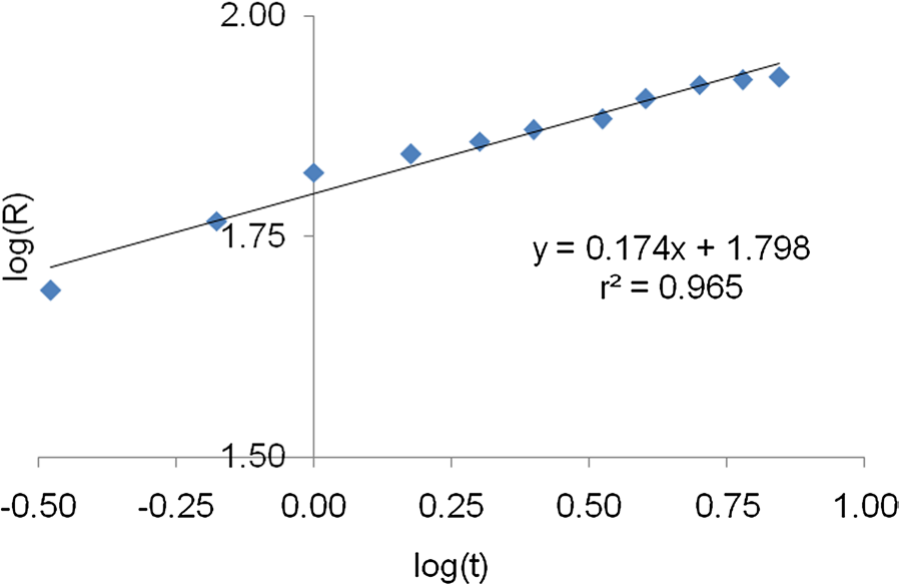
Plot of intraparticle diffusion adsorption kinetic model for activated carbon from rice husk with zinc chloride activation

Model of Lagergren (Table 1) is known as the pseudo first order kinetic expression. The plots of log (q_e_ − q_t_) versus t clearly illustrates a very good correlation with a coefficient value of r^2^ in the rage of 0.974 (Fig. 9). k_1_ and q_e_ are calculated from the slope and intercept of the plot, respectively. The rate constants of first order kinetic models for different types of activated carbon are presented in Table 1.

**Table 1:** The adsorption constant rate of different kinetic models at 0.01 g of the activated carbon dosage and 16 mg L^−1^

Pseudo first-order kinetic model $\log (q_{e} - q_{t} ) = \log (q_{e} ) - \frac{{k_{1} }}{2.303}t$		Pseudo second-order kinetic model $\frac{t}{{q_{t} }} = \frac{1}{{k_{2} q_{e}^{2} }} + \frac{t}{{q_{e} }}$		Intra particle diffusion kinetic model log(R) = log(k_id_) + a log(t)		
k_1_	r^2^	k_2_	r^2^	k_id_	a	r^2^
0.0046	0.974	0.0415	0.998	62.80	0.174	0.965

q_e_ = uptake by the activated carbon at equilibrium, mg g^−1^; q_t_ = uptake by the activated carbon at time t, mg g^−1^; k_1_ = rate of constant of pseudo first order adsorption, min^−1^; k_2_ = rate of constant of pseudo second order sorption, g mg^−1^ min^−1^; R = the fraction solute adsorbed; t = contact time; a = gradient of linear plots and depicts the adsorption mechanism; K_id_ = intra-particle diffusion rate constant (h^−1^)

#### Pseudo second-order kinetic model

**Fig. 10:**
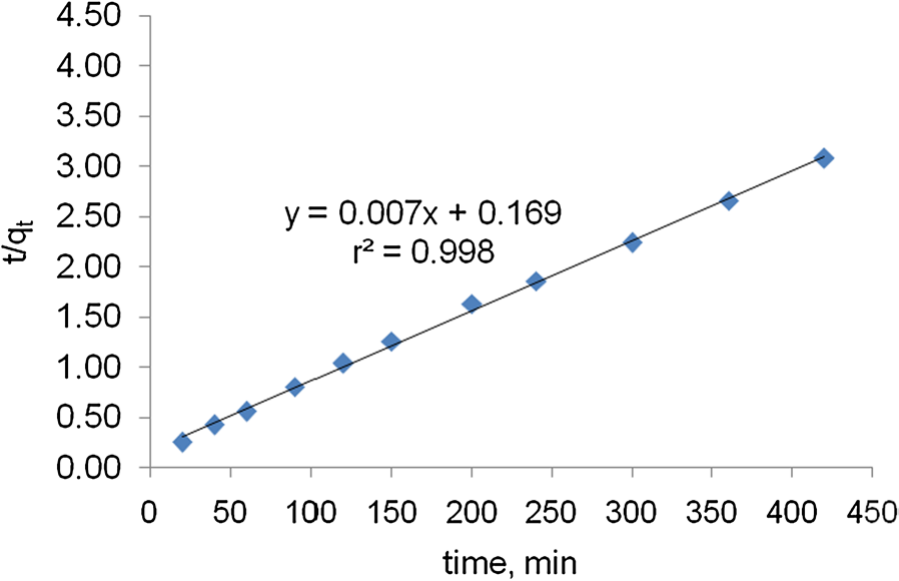
Plot of second order kinetic model for activated carbon from rice husk with zinc chloride activation

In order to differentiate the kinetics of second order rate expressions on the sorbent concentration from the models and on solute concentration, a pseudo second order rate expression was used to evaluate the adsorption kinetic of activated carbon. The plots of t/q_t_ versus t clearly illustrate a very good correlation with a coefficient value of r^2^ in the rage of 0.998 (Fig. 10). The rate constants of pseudo order kinetic models for different types of activated carbon are presented in Table 1.

#### Intra-particle diffusion

**Fig. 11:**
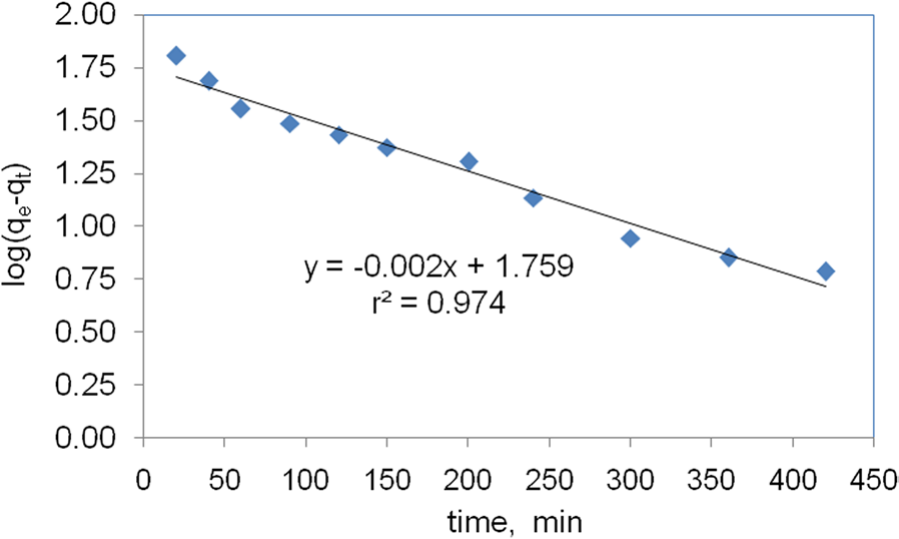
Plot of first order kinetic model for activated carbon from rice husk with zinc chloride activation

The adsorbate species are most probably transported from the bulk of the solution into the solid phase through intra-particle diffusion process, which is often the rate—limiting step in many adsorption process. The possibility of intra-particle diffusion is explored by using the intra-particle diffusion model (Table 1). The plots of log (R) versus log(t) clearly illustrates a very good correlation with a coefficient value of r^2^ in the rage of 0.965 (Fig. 11). The rate constants of intra-particle diffusion kinetic model for different types of activated carbon are presented in Table 1.

### Adsorbent dosage study

**Fig. 12:**
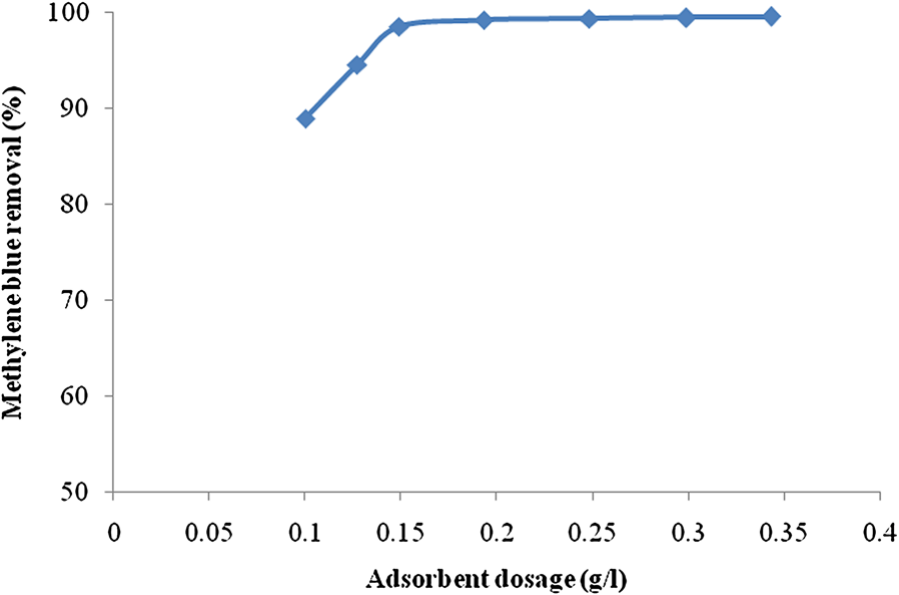
Effect of adsorbent dose of methylene blue removal for activated carbon from rice husk with zinc chloride activation

This study investigates the effect of adsorbent dosage on MB uptake of the kinds of activated carbon by varying the adsorbent dosage. The results of adsorbent dosage study are illustrated in Fig. 12. The results reveal that there is variation of dye removal with respect to dosage. The equilibrium condition of activated carbon is found at the dosage of 0.149 g L^−1^ of activated carbon from rice husk for 10^−4^ M (32 mg L^−1^) concentration of dye. The removal efficiency increases with increase in dosage of adsorbent.

### Study of adsorption isotherm models

Adsorption isotherm provides useful information to estimate the performance of a respective carbon in a full-scale process stream. The isotherms help to determine the possibility to reach a desired purity level with activated carbon treatment. Also they help to calculate the loading of activated carbon at equilibrium, which is a major impact on process economics. Adsorption equilibrium provides fundamental physiochemical data for evaluating the applicability of sorption process as a unit operation. To have a quantitative means of comparing adsorption strength and to design adsorption process effectively, it is useful to use mathematical models for predicting the adsorption. The adsorption capacity depends on the chemical and physical properties of the adsorbent, in which the porosity is one of the important factors. The Freundlich (reported in Ofomaja 2010; Kannan and Veemaraj 2009; Theivarasu et al. 2011), Langmuir (1918) adsorption isotherms (reported in Maiti 2007; Theivarasu et al. 2011; Safa and Bhatti 2011), and Langmuir and Hinshelwood model (reported in Guettai and Amar 2005) are used to analyze the results in describing adsorption behavior of the adsorbent.

#### Freundlich isotherm model

The Freundlich isotherm model is defined by an equation (Table 2). The Freundlich constant k_f_ and n are to be calculated from the intercept and slope of the equation. The constant values of the Freundlich isotherm model are shown in Table 2. The isotherm model curves followed linear equation with correlation coefficient in the range of 0.842.

**Table 2:** Adsorption characteristics of activated carbon prepared from rice husk

Freundlich isotherm constant $\ln (q_{e} ) = \ln (k{}_{f}) + \frac{1}{n}\ln (C_{e} )$			Langmuir isotherm constant $\frac{1}{{q_{e} }} = \frac{1}{{K_{L} }} + \frac{1}{{K_{L} b}} \times \frac{1}{{C_{e} }}$			Langmuir–Hinshelwood isotherm constant $\frac{{C_{e} }}{{q_{e} }} = \frac{1}{{Q_{\hbox{max} } K_{ads} }} + \frac{{C_{e} }}{{Q_{\hbox{max} } }}$		
k_**f**_	n	r^2^	K_L_	B	r^2^	Q_max_	b	r^2^
208.93	3.215	0.842	333.33	3.000	0.940	301.18	3.690	0.995

q_e_ = uptake by activated carbon at equilibrium, mg g^−1^; C_e_ = concentration of solution at equilibrium, mg L^−1^; n = Freundlich constant; k_f_ = adsorption coefficient, L g^−1^; K_L_ = Langmuir adsorption constant, mg g^−1^; b = constant related to energy of adsorption, L g^−1^; Q_max_ = the maximum adsorbed quantity, mole g^−1^; K_ads_ = Langmuir adsorption constant related to energy of adsorption, L M^−1^

#### Langmuir isotherm model

The Langmuir isotherm model is defined by an equation (Table 2). The constant K_L_ and b are calculated from the intercept and slope of the equation. The isotherm model curves of activated carbons followed a linear equation with very good correlation coefficient in the range of 0.940. Langmuir isotherm shows better correlation compared to the Freundlich plot.

#### Langmuir–Hinshelwood plot

The Langmuir and Hinshelwood model can be expressed by an equation (Table 2). The constant values of the Langmuir–Hinshelwood isotherm model are shown in Table 2. The isotherm model curves of activated carbons followed linear equation with very high correlation coefficient in the range of 0.995. Langmuir–Hinshelwood isotherm shows better correlation compared to the Freundlich and Langmuir plots.

**Fig. 13:**
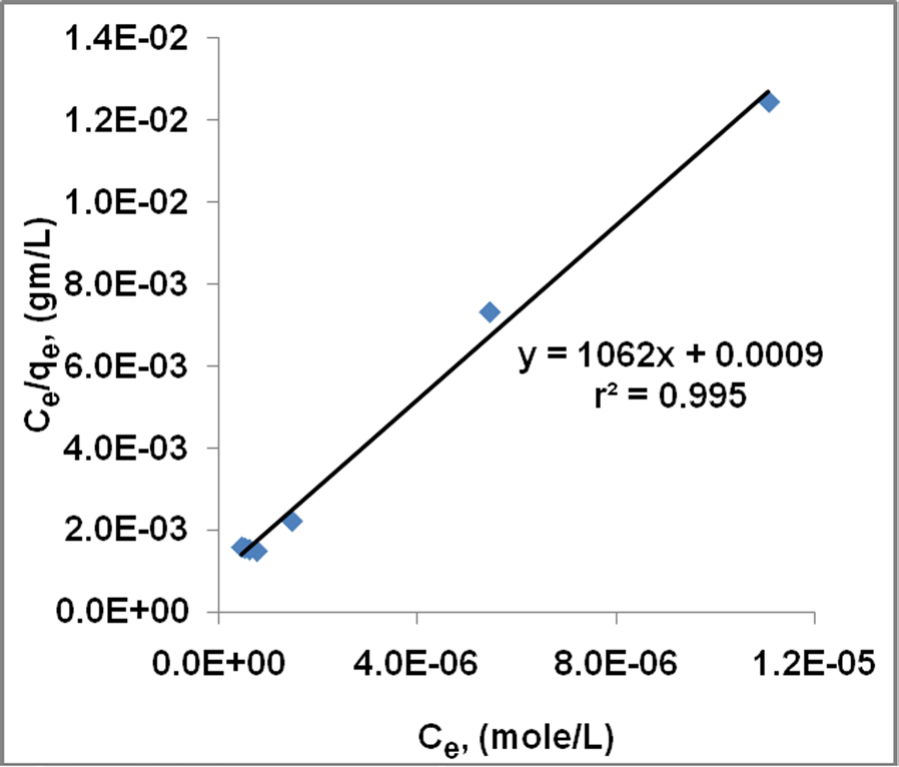
Langmuir–Hinshelwood plot for activated carbon from rice husk with zinc chloride activation

The surface area of the activated carbon is calculated by using the constant value obtained from Langmuir–Hinshelwood plot (Fig. 13). Table 2 shows the calculation of specific surface area of the activated carbon using the Q_max_ value. The specific surface area is estimated to be 737 m^2^ g^−1^ of carbon which indicates a quite satisfactory level of quality of activated carbon. It is mentioned that this area is calculated based on the area covered by MB adsorption which is an indicator of meso-porous area. If the area is calculated by using liquid nitrogen adsorption methods then this would give higher value.

## Conclusion

Activated carbon is an essential material for various industrial usages making environment safe. Agro-waste could be a source material for activated carbon. This study reveals that the porous bio-char can be prepared by leaching ash from rice husk char at low temperature instead of high temperature application. Furthermore, pore structure of porous bio-char increases with increase in temperature of heat treatment. Rice husk char contains a huge percentage of ash that retards the porousness. The study shows that the ash can be removed by alkali treatment. The alkali treated rice husk char develops porousness at intermediate level, further, the pore structure alkali leached rice husk char could be developed with chemical agent. The results also reveal that activated carbon and porous carbon can be prepared with double crucible method to ensure oxygen free or less oxygen environment in the furnace instead of application of inert media. This study shows a method and possibility of activated carbon from agro-waste, and it could be scaled up for commercial production.

## Abbreviations

MB: methylene blue; SEM: scanning electron microscopy; RHC: rice husk char; UV: ultra-violet

### List of symbols

°C: degree Celsius
cm: centimeter
g: gram
h: hour
L: liter
M: Molar
m: meter
mg: milligram
mL: milliliter
